# Hepatic Artery Aneurysm in the Setting of Acute Pancreatitis and Giant Cell Arteritis

**DOI:** 10.7759/cureus.5410

**Published:** 2019-08-17

**Authors:** Levonti Ohanisian, David Rubay, Megan L Morrow, Garrett Basich, Miguel Lopez-Viego

**Affiliations:** 1 Orthopaedic Surgery, Charles E. Schmidt College of Medicine, Florida Atlantic University, Boca Raton, USA; 2 Surgery, Charles E. Schmidt College of Medicine, Florida Atlantic University, Boca Raton, USA; 3 Miscellaneous, St. Mary's College of California, Moraga, USA; 4 Surgery, Bethesda Hospital, Boynton Beach, USA

**Keywords:** hepatic artery, hepatic artery aneurysm, visceral artery aneurysm

## Abstract

Visceral artery aneurysms are rare with an incidence of 0.1%-0.2%. Of these, 20% are hepatic artery aneurysms (HAAs). Despite the potential of remaining asymptomatic for long periods of time, the risk of rupture for HAAs is 20%-80%. Treatment includes operative management with open or endovascular techniques. HAA in the setting of pancreatitis has been reported in two prior cases outside of the United States. However, there have been no cases describing the association of HAA and giant cell arteritis (GCA). We present a rare case of an 80-year-old male with a history of GCA who was found to have developed HAA following an episode of acute pancreatitis that was repaired surgically with an open technique. To our knowledge, the association between HAA with acute pancreatitis and GCA has not been reported before.

## Introduction

Visceral artery aneurysms are rare, with an incidence of 0.1%-0.2% [1]. Hepatic artery aneurysms (HAAs) were first described by Wilson in 1809 [2] and represent 20% of all visceral aneurysms [1]. Although many HAAs remain asymptomatic for long periods of time, the risk for rupture ranges between 20%-80% with mortality rates as high as 21% [3-4]. Risk factors include arteriosclerotic disease, fibromuscular dysplasia, cystic medial necrosis, and portal hypertension [5].

Unfortunately, the strength of evidence guiding the timing of repair is lacking. As opposed to the treatment of aortic aneurysms, which are repaired at a diameter of 5.5 cm or growth of over 0.5 cm in six months due to strong data suggesting an increased risk of rupture [6], the risk of rupture relative to the size of an HAA is unknown [7]. Treatment includes operative management with open or endovascular techniques [5]. Elective repair has shown to be a relatively safe intervention. In a retrospective study at the Mayo Clinic spanning 23-years, only one of 21 patients with HAA passed away from an elective HAA repair, a much lower rate than the risk of rupture [5].

HAA in the setting of pancreatitis has been reported in two prior cases outside of the United States [8-9]. However, there have been no cases describing the formation of HAA in a patient with giant cell arteritis (GCA). We present the case of an 80-year-old male with a history of GCA who was found to have developed an HAA following an episode of acute pancreatitis that was repaired surgically with an open technique.

## Case presentation

An 80-year-old male with a past medical history significant for renal calculi, GCA, atrial fibrillation, and a 68 pack-year history of smoking presented with epigastric pain and was found to have acute pancreatitis. Full workup was negative, and the etiology of the pancreatitis was concluded to be idiopathic. Notably, the computed tomography (CT) scan demonstrated a 3.2 cm pancreatic cyst at the tail of the pancreas. Furthermore, there were no abnormalities noted in the abdominal vasculature at this time.

**Figure 1:**
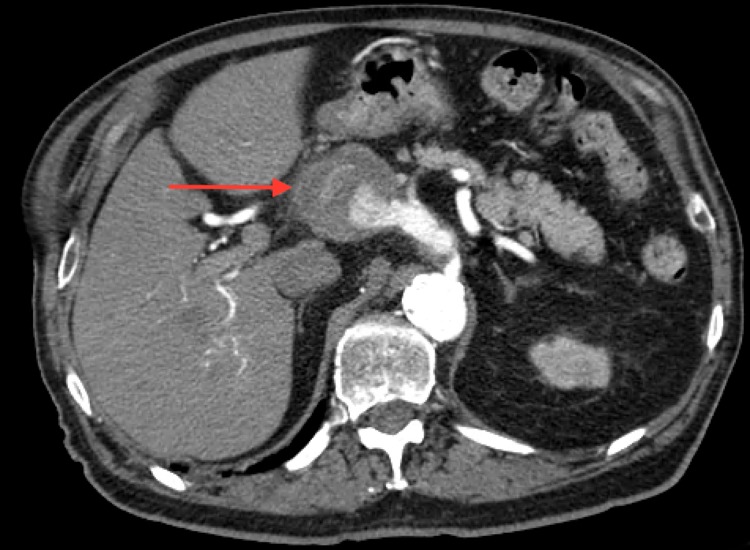
Computed tomography (CT) of the abdomen illustrating 5.1 cm hepatic artery aneurysm

Endoscopic ultrasonography (EUS) with fine-needle aspiration (FNA) showed the cyst to have benign features. Follow-up imaging in six months was done using a CT scan instead of magnetic resonance imaging (MRI) due to the patient’s incompatible hip prostheses. The CT scan revealed regression of the pancreatic cyst to 1.5 cm. Additionally, a 2.5 cm HAA was discovered. The patient was referred to vascular surgery but delayed follow-up. One month later, the patient experienced another episode of epigastric pain. The CT scan did not demonstrate any findings of pancreatitis but did illustrate an HAA with a diameter of 3.2 cm. The patient chose to seek a second opinion and presented back to the clinic the following month. CT scan at this time demonstrated aneurysmal growth to 5.1 cm (Figure 1).

**Figure 2:**
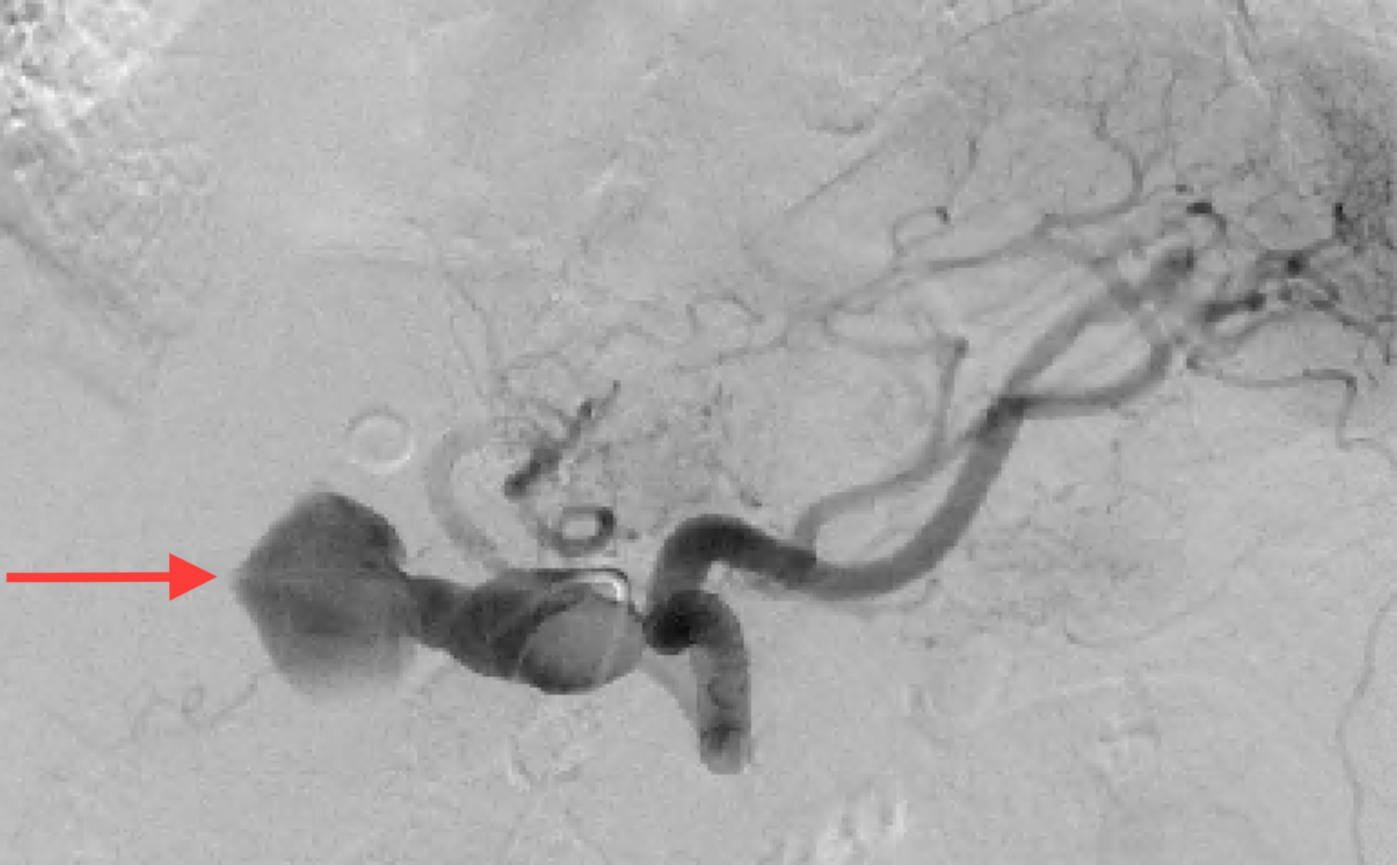
Aortogram with selective celiac arteriography demonstrating an enlarged fusiform common hepatic artery aneurysm

**Figure 3:**
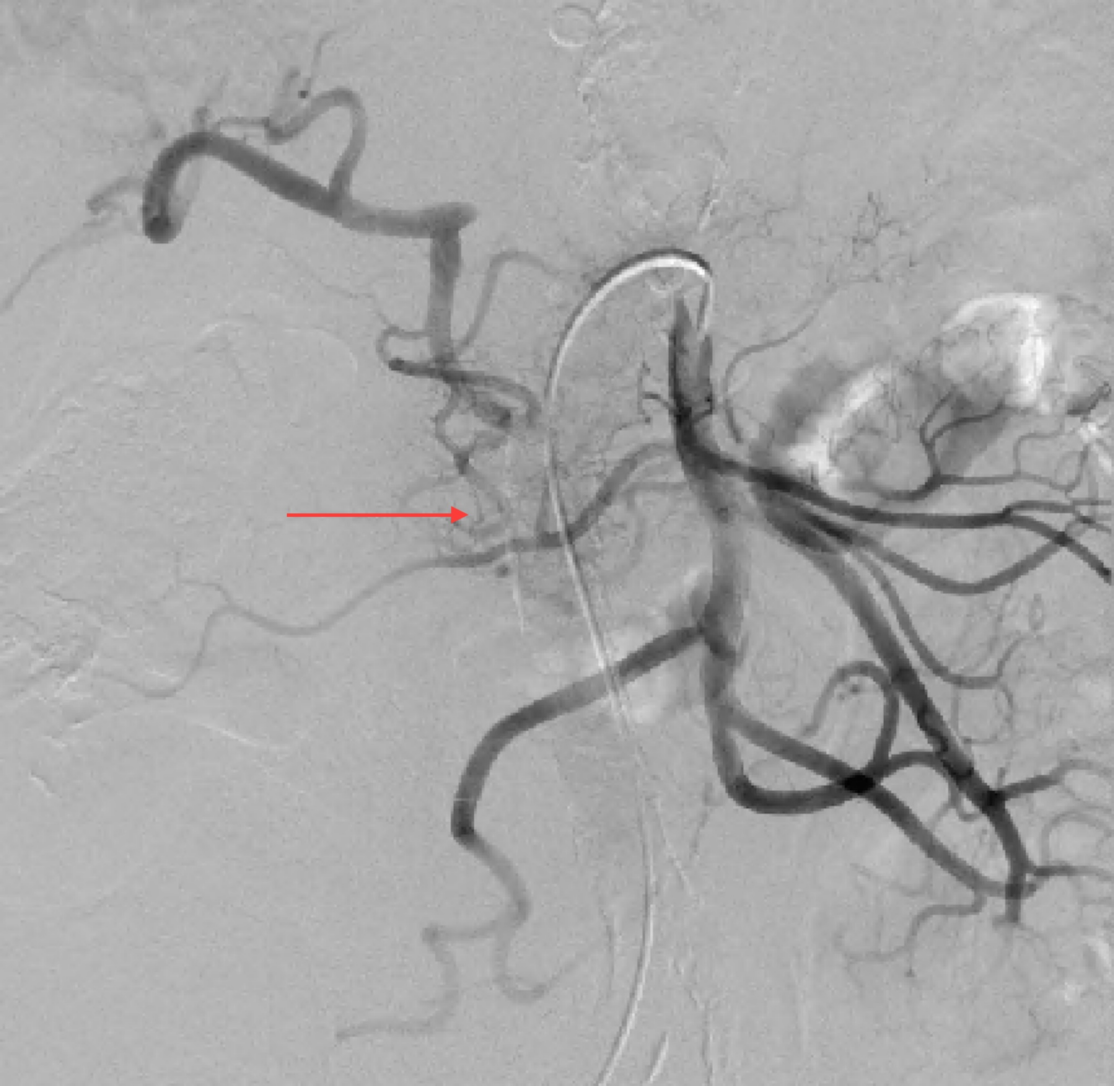
Aortogram with superior mesenteric arteriography demonstrating reconstitution of the proper hepatic artery via retrograde flow in the gastroduodenal artery

Aortogram with selective celiac and superior mesenteric arteriography demonstrated an enlarged fusiform common HAA (Figure 2). The common hepatic artery was noted to be aneurysmal from its origin to where it occludes distally with reconstitution of the proper hepatic artery via retrograde flow in the gastroduodenal artery (GDA) from the superior mesenteric artery (SMA) (Figure 3). Of note, the celiac artery and splenic artery did not appear aneurysmal, and the left gastric artery appeared to come off the splenic artery distal to the origin of the aneurysmal common hepatic artery.

Due to the distal reconstitution of retrograde flow into the GDA from the SMA, and left gastric artery coming off of the splenic artery distal to the origin of the aneurysmal common hepatic artery, the patient was taken to the operating room for open HAA repair. An upper midline laparotomy was performed. The stomach was found to be displaced inferiorly secondary to the aneurysm. The hepatic artery was found to be aneurysmal while the celiac, splenic, and left gastric arteries were of normal caliber. The celiac, splenic, and left gastric artery were isolated and controlled. The distal aspect of the aneurysm was palpated and found to be thrombosed. The celiac artery was clamped, and an arteriotomy was made which created an opening of the aneurysmal sac where significant amounts of mural thrombus were found and evacuated. Afterward, the aneurysmal common hepatic artery was isolated and the celiac trunk was reconstructed with pledgeted prolene sutures. Finally, arterial blood flow through the splenic, left gastric, and both left and right hepatic arteries was confirmed using intraoperative Doppler.

## Discussion

HAAs are rare [1]. The epidemiology of this pathology involves patients in their fifth and sixth decades of life and may present with epigastric pain or right upper quadrant pain that radiates to the back [10]. Often, HAAs are asymptomatic but can present catastrophically on rupture [10-12]. Angiographic studies should be obtained in order to evaluate for additional lesions seen in up to 20% of cases [13] as well as to evaluate for collateral pathways, the circulation to the liver, pancreas, spleen, and stomach, as well as the extent of involvement of the celiac artery trunk. Furthermore, angiography provides information regarding the feasibility of aneurysmal repair as opposed to arterial ligation.

Unfortunately, the strength of evidence guiding when to repair these aneurysms is lacking and the risk of rupture relative to the size of an HAA is unknown [7]. Treatment may include operative management with open or endovascular techniques [5] and is considered a relatively safe intervention. In a retrospective review of 21 HAAs at the Mayo Clinic over a 23-year span, the authors concluded that endovascular therapy may be an effective and innovate way of treating HAA, however, treatment should be tailored to each patient’s unique circumstance [5]. Specifically, lesions that are isolated to the common hepatic artery may be coil embolized only if there is collateral flow to the proper hepatic artery through the GDA [5]. Only one of 21 patients in their series passed away from an elective HAA repair, a much lower rate than the risk of rupture, which may total 40% [5].

Due to the size of our patient’s aneurysm and the rapid rate of progression, we elected to intervene using an open technique. Due to the local anatomy of the celiac trunk, preoperative analysis of collateral blood supply to the liver, stomach, pancreas, and spleen are of vital importance. For example, our patient had distal reconstitution of the proper hepatic artery through retrograde flow into the GDA from the SMA, and the left gastric artery came off of the splenic artery distal to the origin of the aneurysmal common hepatic artery on mesenteric arteriography. Therefore, the patient was treated with open HAA repair by ligating the common hepatic artery.

Risk factors for HAA include smoking, arteriosclerotic disease, fibromuscular dysplasia, cystic medial necrosis, and portal hypertension [5]. The patient in our case had a medical history of GCA, a systemic vasculitis that affects both medium and large arteries with peak age of diagnosis approximately at the age of 80 [14]. Complications revolve around the sequelae of arterial wall inflammation and commonly include vision loss, with 15% of patients resulting in permanent vision loss [15]. High-dose glucocorticoids are the mainstay of treatment [14]. The pathophysiology of GCA involves a dysregulated interaction between the arterial walls of medium and large-sized arteries and the innate and adaptive immune system [14]. This results in local vascular damage triggered by toll-like receptors activating vascular dendritic cells, endothelial cells, smooth muscle cells, and fibroblasts that all interact with T cells (predominantly CD4+) and macrophages leading to the subsequent vascular pathology [16-17].

GCA is an independent risk factor for the development of aortic aneurysms, and extracranial manifestations of GCA are reported to occur in approximately 30%-80% of cases [18]. In fact, aortic aneurysms are two times more likely in patients with GCA than in the general population [18]. Despite this increased risk for aortic aneurysms, to our knowledge, there have been no reports of patients with GCA presenting with isolated HAAs.

Acute pancreatitis has been linked to both HAAs and pseudoaneurysms [19,8-9]. Yu et al. presented a case of a 61-year-old woman who presented with pancreatitis who developed a hepatic artery pseudoaneurysm on CT imaging eight days later than was not present on previous imaging [19]. Zabicki et al. studied pancreatitis-related pseudoaneurysms of visceral vessels in a retrospective clinical study and concluded that the most common artery affected was the splenic artery (46.7%), common hepatic artery (13.3%) and right gastroepiploic artery (13.3%) [20]. The development of GDA aneurysms has also been associated with acute pancreatitis. To our knowledge, our case is one of two other cases reported of HAAs developing after an episode of acute pancreatitis [8-9]. Possible pathophysiological explanations include pancreatic enzyme induced inflammation leading to lesions in peripancreatic arteries [8]. In the setting of compromised medium and large vessel vasculature, such as in our patient with GCA, acute pancreatitis may be the trigger for aneurysm development in susceptible arteries.

The significance of our case is that it represents another severe sequela of acute pancreatitis - HAA formation. Furthermore, it may present GCA as an additional risk factor for HAA formation. To our knowledge after a thorough literature review, we present a third case of HAA seen after an episode of acute pancreatitis. Despite our unique case, there is still a scarcity of such cases in the literature and future case reports and retrospective studies are needed in order to fully elucidate the etiology, risk factors, and pathogenesis of HAA formation in relation to acute pancreatitis and GCA.

## Conclusions

HAAs are rare. Traditional risk factors include smoking, arteriosclerotic disease, fibromuscular dysplasia, and cystic medial necrosis. Treatment includes operative management with open or endovascular techniques. Preoperative angiography is essential in assessing for collateral pathways, the circulation to the liver, pancreas, spleen, and stomach as well as the extent of involvement of the celiac artery trunk. To our knowledge, this is the third case of HAA after an episode of acute pancreatitis. We conclude that HAAs should be on the differential diagnosis of a patient with a history of acute pancreatitis. Additionally, HAAs may be a sequela of acute pancreatitis and GCA, although further research is needed to corroborate these findings.
